# Morphine decreases the pro-angiogenic interaction between breast cancer cells and macrophages *in vitro*

**DOI:** 10.1038/srep31572

**Published:** 2016-08-12

**Authors:** Samira Khabbazi, Zeyad D. Nassar, Yannick Goumon, Marie-Odile Parat

**Affiliations:** 1 University of Queensland School of Pharmacy, PACE, 20 Cornwall Street. Woollloongabba QLD 4102, Australia; 2 CNRS UPR3212, Institut des Neurosciences Cellulaires et Intégratives, Centre National de la Recherche Scientifique and University of Strasbourg, 5 rue Blaise Pascal, 67084 Strasbourg, France

## Abstract

Interactions between the various cell types that constitute a solid tumour are essential to the biology of the tumour. We evaluated the effect of morphine on the proangiogenic interaction taking place between macrophages and breast cancer cells *in vitro*. The conditioned medium (CM) from breast cancer cells co-cultured with macrophages elicited endothelial cell proliferation and tube formation. This effect was inhibited if the co-culture occurred in the presence of morphine. The CM from breast cancer cells or macrophages grown individually, whether or not prepared in the presence of morphine, was ineffective in stimulating EC proliferation or tube formation. Using a mouse antibody array, we identified several angiogenesis-regulating factors differentially expressed in the CM of co-cultured cells prepared in the presence or absence of morphine, amongst which interleukin (IL)-6, tumour necrosis factor (TNF)-α and vascular endothelial growth factor (VEGF)-A. VEGF was induced in both cell types by the co-culture and this was prevented by morphine in a non-naloxone reversible fashion. The effect of CM from co-cultured cells on endothelial tube formation, but not proliferation, was prevented by anti-VEGF neutralizing antibody. Our results indicate that morphine prevents, in part via modulating VEGF-A expression, the pro-angiogenic interaction between macrophages and breast cancer cells.

In addition to the uncontrolled proliferation of cancer cells, components of the microenvironment, including non-malignant cells such as tumour associated macrophages (TAMs), endothelial cells and fibroblasts, play a key role in promoting tumour development. Within the tumour, paracrine interactions between stromal and cancer cells exacerbate the local inflammatory, pro-metastatic and pro-angiogenic features of the microenvironment^1^. Vascular endothelial growth factor (VEGF)-A is a master regulator of normal and pathological angiogenesis that acts on endothelial cell (EC) receptors to promote vascular permeability and EC survival, proliferation, migration and tube formation^2^. Upregulation of VEGF-A expression is primarily stimulated by hypoxia via the hypoxia-inducible factor (HIF)^3^. Both cancer and stromal cells^4^ can respond to hypoxia by overexpression of VEGF and other hypoxia-regulated genes. In addition, VEGF-A gene expression is also regulated by oncogenic mutations, and by cytokines and other growth factors. Specifically, it has been shown that interaction between stromal cells and cancer cells can increase VEGF expression in normoxic conditions^5^,^6^.

Morphine is administered to cancer patients, either to manage the perioperative pain associated with tumour ablation, or to control cancer-induced pain. Opioids are documented to play a role in the regulation of tumour growth and metastasis, without a clear consensus on whether their net effect is beneficial or deleterious^7^. The discrepancy between studies may stem from the use of different models, a vast range of opioid dose and concentrations in chronic or acute regimens, and actions on opioid as well as non-opioid receptors^8^. It is clear that opioids regulate a number of processes involved in tumour growth and metastasis, including cancer cell proliferation and survival, immune surveillance and angiogenesis (reviewed in^7^). In this context, published data on the effect of morphine on tumour angiogenesis in mouse models are particularly interesting. Morphine was initially found to be pro-angiogenic in a model involving human MCF7 breast cancer cells injected in the mammary fat pad of immuno-compromised mice^9^ and this was later confirmed in a syngeneic breast tumour model^10^, then in a transgenic mouse spontaneously developing breast carcinoma^11^. The three studies employed low, sub-analgesic doses of morphine. Diametrically different results were obtained using Lewis lung carcinoma cells implanted subcutaneously with matrigel in athymic mice and high doses of morphine^12^,^13^. Interestingly, one proposed mechanism was the inhibition by morphine of tumour leucocyte infiltration^13^. Recently, morphine administered at analgesic doses to two models of breast tumour-bearing mice did not affect tumour growth or angiogenesis^14^.

Morphine has been documented to inhibit hypoxia-induced VEGF expression in endothelial and myocardial cell lines (*in vitro*) and in a rat coronary ligation model of myocardial ischemia (*in vivo*)^15^,^16^. We previously showed that morphine modulates the paracrine interaction between breast cancer cells and stromal cells in co-culture models; the production of matrix metalloprotease (MMP)-9 and the expression of macrophage alternative activation markers were decreased if the co-culture occurred in the presence of morphine^17^. In the present study, we hypothesized that morphine decreases the pro-angiogenic interaction between breast cancer cells and macrophages. We investigated the effect of morphine on the production of proangiogenic factors and functional consequences resulting from the paracrine communication between RAW264.7 macrophages and 4T1 breast cancer cells.

## Results

### Morphine prevents co-culture CM-induced proliferation and tube formation of endothelial cells

**Figure 1:**
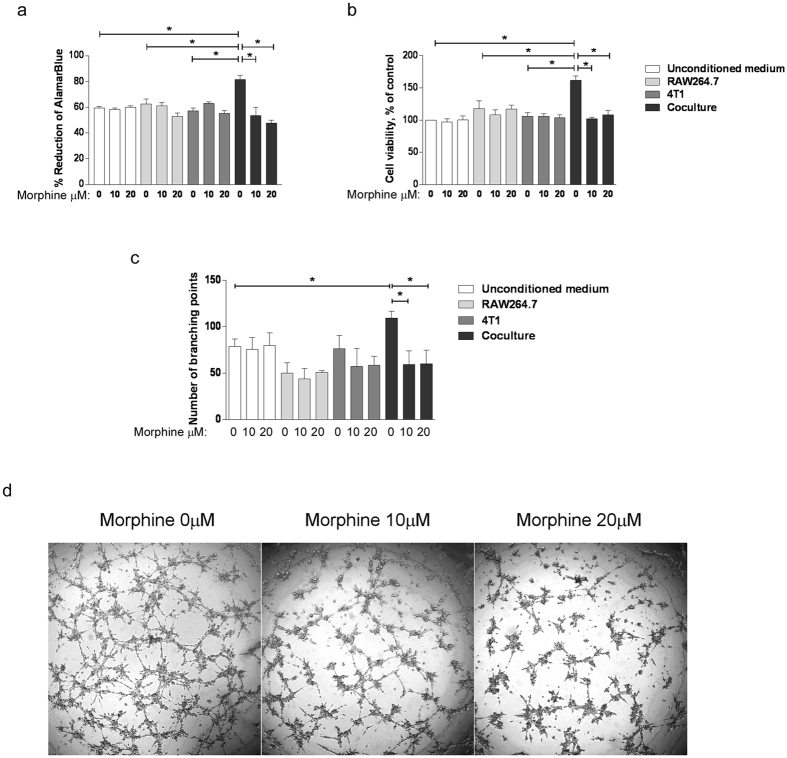
Morphine prevents the ability of CM from co-cultured cells to elicit EC proliferation and tube formation. (**a**) Determination of BAEC proliferation using the AlamarBlue assay. BAEC were exposed for 48 h to the conditioned media of 4T1, RAW264.7 cells grown alone or together in the presence (10 or 20 μM as indicated) or absence of morphine. Cells were added with AlamarBlue Reagent and incubated for 4 h. The absorbance at 570 nm and 600 nm was recorded. Cell viability is presented as the percentage of AlamarBlue reagent reduction. Mean ± S.D. is shown for N = 3 independent experiments. *P = 0.05. (**b**) Determination of BAEC proliferation using the MTT assay. BAEC were exposed for 48 h to the conditioned media of 4T1, RAW264.7 cells grown alone or together in the presence (10 or 20 μM as indicated) or absence of morphine. Cells were incubated for 5 h in MTT-containing medium. The absorbance at 595 nm was determined. Cell viability is presented as the percentage of viability of control cells. Mean ± S.D. is shown for N = 3 independent experiments. (**c**) BAEC were incubated with either 4T1, RAW264.7 cell-conditioned media or unconditioned media as the control and added onto polymerized matrigel for 6 h. Capillary-like tubes were imaged for quantification. Results are reported as the mean number of branching points ± S.D. N = 3 independent experiments. *P = 0.05. (**d**) Representative images of the capillary-like tubes formed by BAEC.

To examine the effect of the interaction between cancer cells and macrophages on angiogenesis-regulating factors, the 48 h conditioned media of RAW264.7 cells and 4T1 cancer cells grown individually or in transwell co-culture in the presence or absence of morphine were collected and added to endothelial cells. EC proliferation (Fig. 1a,b) and tube formation (Fig. 1c) were measured. The proliferation assay, via either the AlamarBlue (Fig. 1a) or MTT (Fig. 1b) assay, showed that the conditioned media from the co-cultured cells induced a significant increase in EC proliferation at 48 h, compared to unconditioned medium or conditioned medium of cells grown individually. Interestingly the increased proliferation of EC induced by CM from co-cultured cells was prevented if morphine was present during the co-culture (Fig. 1a,b). Morphine added to unconditioned medium did not affect EC proliferation. Further to EC proliferation, EC capillary like tube formation *in vitro* in response to conditioned media was measured (Fig. 1c,d). Quantification of the number of branching points indicated that the CM from co-cultured cells was more efficient in eliciting tube formation than the CM from RAW264.7 and 4T1 cells grown alone. The tube formation induction by CM from co-cultured cells was abolished if the co-culture was carried out in the presence of morphine (Fig. 1c). These results indicate that morphine modulates the paracrine proangiogenic interaction between macrophages and breast cancer cells.

### Morphine alters the production of proangiogenic factors in the conditioned medium of co-cultured cells

**Figure 2:**
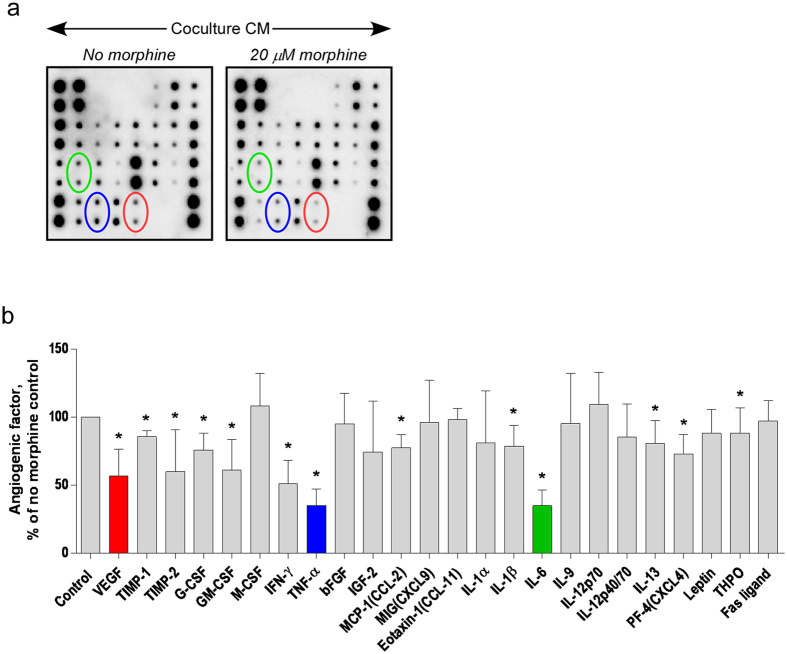
Morphine decreases co-culture-induced IL-6, TNF-α and VEGF-A production. Conditioned media of 4T1 and RAW264.7 cell co-cultured in the presence (20 μM) or absence of morphine were added to mouse angiogenesis antibody array membranes. (**a**) The images were captured using a ChemiDoc Touch Imaging System (Bio-Rad Laboratories Inc.) Green, Blue and red circling were added to locate duplicate spots corresponding to VEGF, TNF-α and IL-6, respectively. (**b**) Results are normalized to positive control signal intensities and angiogenic factors expressed as % of the amount measured in the no-morphine control. Graph shows mean ± S.D. (N = 3 separate experiments, each performed with a mix of three independent co-culture media). *P = 0.05.

To determine which angiogenic factor(s) in the macrophage and breast cancer cells co-culture CM are affected by morphine, an angiogenesis antibody array membrane was used. Membranes were incubated with CM from cells co-cultured the presence or absence of 20 μM morphine (Fig. 2a). Densitometric analysis showed that the production of several angiogenic factors in co-cultured cell-CM was significantly decreased by morphine (Fig. 2b). Three pro-angiogenic factors were chosen for further analysis: TNF-α and IL-6, because their production was most strongly inhibited by morphine, and VEGF-A, because of its role as a master regulator of angiogenesis.

### Effect of morphine on the production of IL-6, TNF-α and VEGF-A by co-cultured macrophages and breast cancer cells

**Figure 3:**
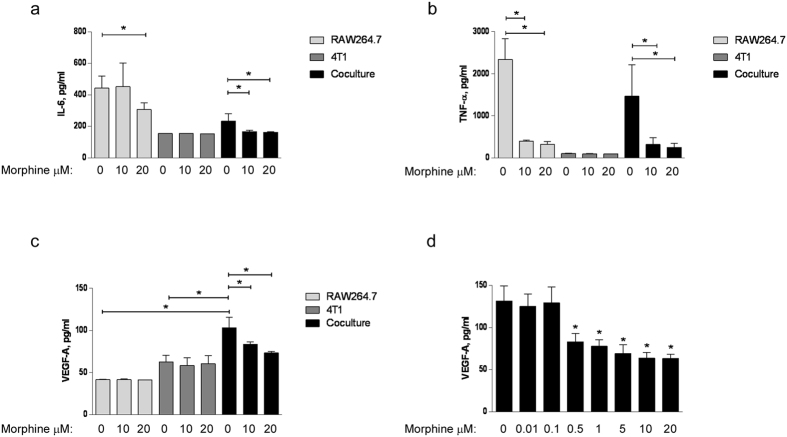
Effect of morphine on the production of pro-angiogenic factors IL-6, TNF-α and VEGF-A. Protein level of (**a**) IL-6, (**b**) TNF-α and (**c**) VEGF–A in the conditioned media of 4T1, RAW264.7 alone or in co-culture in the presence (10 μM and 20 μM) or absence of morphine was measured by ELISA. (**d**) ELISA quantification of VEGF-A present in the conditioned medium of RAW264.7 cells co-cultured with 4T1 in the absence or presence of morphine at indicated concentrations (10 nM, 100 nM, 500 nM, 1 μM, 5 μM, 10 μM and 20 μM). Results are shown as the mean of growth factor concentrations ± S.D. in three independent experiments (N = 3), *P = 0.05.

We assessed the effect of morphine on the production of IL-6, TNF-α and VEGF-A by macrophages and breast cancer cells grown individually or co-cultured in a transwell (Fig. 3). The 48 h CM were collected and tested using ELISA. Control cells were grown individually with or without morphine. The results confirmed that the concentrations of IL-6 (Fig. 3a), TNF-α (Fig. 3b) and VEGF-A (Fig. 3c) were significantly decreased by morphine in the CM from co-cultured RAW264.7 and 4T1 cells. However, the amount of IL-6 and TNF- α was lower in CM from co-cultured cells than in the CM from RAW264.7 cells grown individually, indicating that although they might contribute to it, neither of these cytokines directly mediates the pro-angiogenic effect of the CM from co-cultured cells. In contrast, the interaction of the macrophages and cancer cells resulted in a significant increase in VEGF-A production compared with the CM of cells grown individually (Fig. 3c). Moreover, morphine prevented the increase in VEGF-A production in co-cultured cells while it had no effect on the production of VEGF-A by cells grown individually (Fig. 3c), a pattern reminiscent of the effect of morphine on CM in functional assays (Fig. 1). The concentrations of morphine used in our *in vitro* experiments are in the higher range of concentrations determined in the circulation of cancer patients receiving high doses of morphine^18^. To test the relevance of our findings to patients receiving lower doses of morphine^19^, we examined VEGF-A production in RAW264.7 and 4T1 cells co-cultured in the presence a range of morphine concentrations (10 nM, 100 nM, 500 nM, 1 μM, 5 μM, 10 μM, 20 μM) (Fig. 3d). A statistically significant decrease in VEGF-A production was observed at concentrations of morphine of 500 nM and higher (Fig. 3d).

### Contribution of breast cancer cells and macrophages to the expression of VEGF-A expression and its regulation by morphine

**Figure 4:**
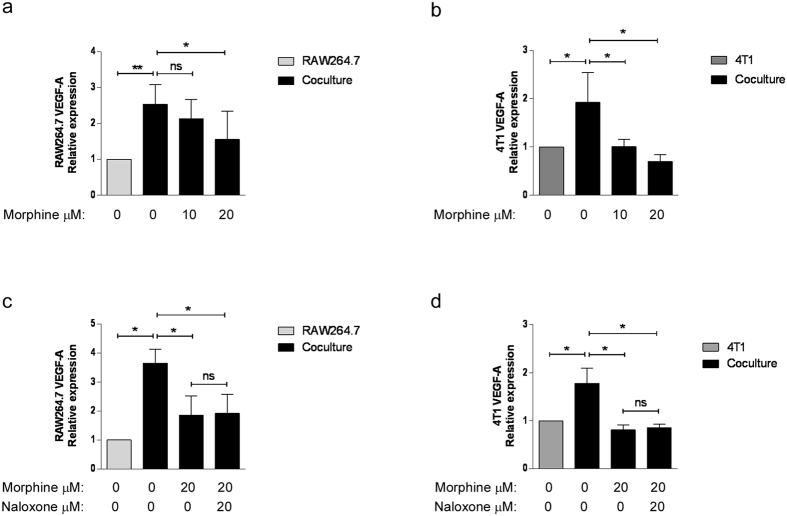
Morphine inhibits induction of VEGF-A mRNA in co-cultured breast cancer cells and macrophages. Quantitative Real-time PCR determination of VEGF-A mRNA levels in (**a**) RAW264.7 cells and (**b**) 4T1 cells co-cultured for 48 h. Results are reported relative to each cell type grown individually. Mean ± S.D. is shown. N = 3–5 independent experiments. *P = 0.05, **P = 0.004. (**c, d**) RAW264.7 macrophages and 4T1 cells were cultured alone or together in the presence of morphine (20 μM) and naloxone (20 μM) as indicated. Quantitative Real-time PCR determination of VEGF-A mRNA levels in (**c**) RAW264.7 cells and (**d**) 4T1 cells. Mean ± S.D. is shown. N = 3 independent experiments. *P = 0.05.

To assess the contribution of each cell type to increased VEGF-A production within the co-culture, and their response to morphine, RNA from breast cancer cells and macrophages was prepared and the expression of VEGF mRNA tested by real time RT-PCR (Fig. 4). Both RAW264.7 (Fig. 4a) and 4T1 cells (Fig. 4b) contribute to increase VEGF-A expression when they are placed in co-culture in a transwell. Interestingly, morphine significantly reduced the co-culture-induced VEGF-A expression by both cell types at mRNA level. To test whether the effect of morphine was opioid receptor-mediated, the cells were incubated in medium containing 20 μM morphine in the presence or absence of the antagonist naloxone at equimolar concentration. The effect of morphine on VEGF mRNA production in transwell co-cultures was not reversed by naloxone in either cell type (Fig. 4c,d).

### VEGF-A mediates the modulatory effect of morphine on EC tube formation but not EC proliferation induced by CM from co-cultured cells

**Figure 5:**
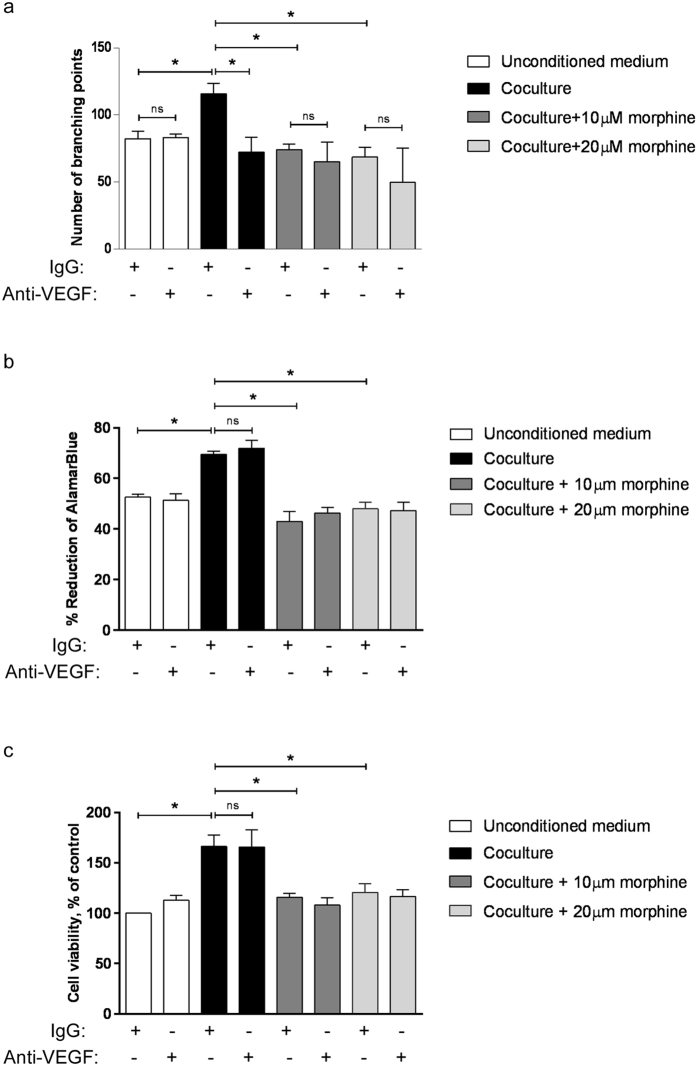
Effect of anti-VEGF antibody on BAEC tube formation and proliferation in response to CM from breast cancer cells and macrophages grown individually or in co-culture. (**a**) BAEC added onto polymerized matrigel and incubated for 6 h with un-conditioned or conditioned media in the presence of anti-VEGF-A antibody or non-immune IgG. Results are shown as the number of branching points. N = 3 independent experiments. *P = 0.05. (**b**) BAEC were exposed for 48 h to the conditioned media of 4T1, RAW264.7 grown individually or co-cultured in the presence or absence of morphine (10 μM and 20 μM). Anti-VEGF antibody or non-immune IgG were added to the medium. Cells then were added with AlamarBlue Reagent and incubated for 4 h. The absorbance at 570 nm and 600 nm was measured. Cell viability is presented as the percentage of AlamarBlue reagent reduction. Mean ± S.D. is shown in N = 3 independent experiments. *P = 0.05. (**c**) BAEC were exposed for 48 h to the conditioned media of 4T1, RAW264.7 grown individually or co-cultured in the presence or absence of morphine (10 μM and 20 μM). Anti-VEGF antibody or non-immune IgG were added to the medium. Cells were incubated for 5 h in MTT-containing medium. The absorbance at 595 nm was determined. Cell viability is presented as the percentage of viability of control cells. Mean ± S.D. is shown for N = 3 independent experiments. *P = 0.05.

To determine whether the effect of morphine on CM-mediated EC proliferation and tube formation is VEGF-A mediated, we tested EC proliferation and *in vitro* tube formation in response to CM from co-cultured cancer cells and macrophages in the presence of neutralizing anti-mouse VEGF antibody. The effect of the neutralizing antibody was compared to that of non-immune IgG. The neutralizing anti-VEGF-A antibody prevented the increased tube formation induced by CM from co-cultured cells, bringing it down to the level observed with CM of co-cultured cells prepared in the presence of morphine (Fig. 5a). The neutralizing antibody had no effect on tube formation in un-conditioned medium or in CM from breast cancer cells or macrophages co-cultured in the presence of morphine (Fig. 5a). These results suggest that decreased VEGF-A production may mediate the effect of morphine on decreased CM-induced tube formation. In contrast, the neutralizing anti-VEGF-A antibody had no effect on the increased EC cell proliferation induced by the CM from co-cultured cells (Fig. 5b,c). This indicates that the EC proliferation induced by CM from co-cultured cells is mediated by other pro-angiogenic factor(s) whose production is increased when paracrine interaction takes place between macrophages and tumour cells, and down-regulated by morphine.

### The production of VEGF-A in CM from co-cultured cells is secondary to that of IL-6 but not TNF-α

**Figure 6:**
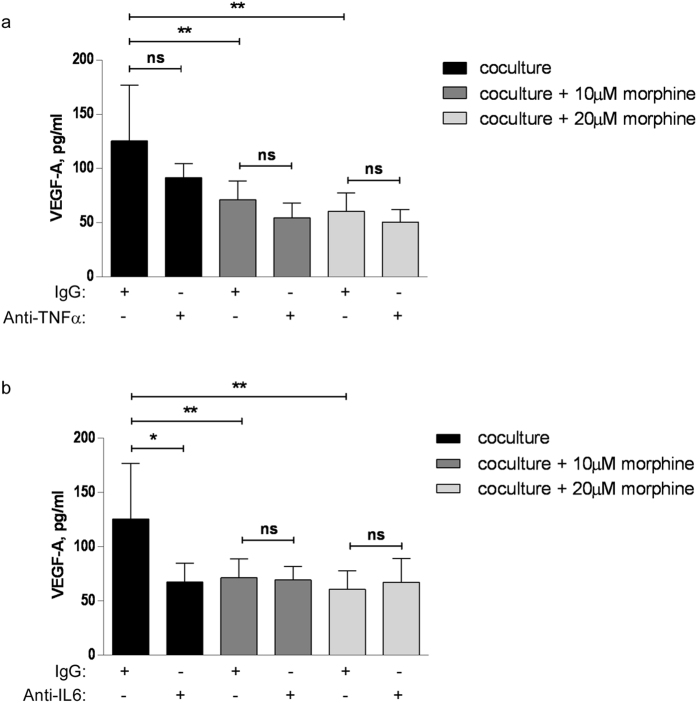
Effect of anti-TNF-α or anti-IL-6 antibody on VEGF production in the CM of breast cancer cells and macrophages in co-culture. (**a**) 4T1 and RAW264.7 cells were grown in co-culture in the presence of anti-TNF-α antibody or non-immune IgG and added or not with morphine. N = 3–7 independent experiments. **P < 0.01. (**b**) 4T1 and RAW264.7 cells were grown in co-culture in the presence of anti-IL-6 antibody or non-immune IgG and added or not with morphine. N = 4–7 independent experiments. *P < 0.05, **P < 0.01.

Hypoxic-response element-independent induction of VEGF production in cancer cells in response to IL-6 has been documented^20^. Similarly, TNF-α has been reported to induce VEGF production in macrophages^21^. To test whether the effect of morphine on VEGF production might be secondary to its effect on IL-6 and/or TNF-α, we assessed whether anti-IL-6 or anti-TNF-α neutralising antibodies altered the concentration of VEGF in the conditioned medium of co-cultured cells. To that extent, co-culture of breast cancer cells and macrophages was carried out with neutralizing antibody or non-immune IgG, in the presence or absence or morphine. The anti-TNF-α antibody did not significantly alter the co-culture-induced increase in VEGF-A production despite a trend towards decreased concentrations of VEGF for each morphine concentration (Fig. 6a), indicating that the effect of morphine on VEGF production is not likely to be mediated by the decrease in TNF-α production. In contrast, the anti-IL-6 antibody neutralized the co-culture induced increase in VEGF concentration in the conditioned medium, and morphine had no further effect than the antibody, in favour of morphine-induced reduction in IL-6 mediating the morphine-induced decrease in VEGF production (Fig. 6b).

## Discussion

Hypoxia is a major inducer of VEGF expression and occurs when the tumour reaches a size such that tumour cells in the center exceed in distance from the nearest capillary the limit of oxygen diffusion (100–200 μm)^22^. It has been previously shown that morphine prevented hypoxia-induced VEGF production by Lewis lung carcinoma (LLC) cells *in vitro* via inhibition of nuclear localization and DNA binding of the hypoxia-induced factor HIF-1α^12^.

Experiments conducted in “normoxia” show that morphine prevents the induction of VEGF production in cultured RAW264.7 macrophages exposed to lipopolysaccharide (LPS)^23^. This is in agreement with our results. In our experiments, induction of VEGF occurs at transcriptional level when breast cancer cells and macrophages are placed in separate chambers and allowed to interact in a paracrine manner. Our results show that both cell types contribute to the increase in VEGF in the conditioned medium that they share, and that both cell types respond to morphine by decreasing VEGF mRNA synthesis. That morphine can decrease hypoxia-independent VEGF induction complements its hypoxia-dependent effects in tumours with hypoxic regions, and may further be relevant to the biology of micrometastases, where the center of the tumour mass is not yet hypoxic.

This novel effect of morphine in moderating the paracrine reciprocal stimulation between breast cancer cells and stromal cells adds to previously documented actions for this opioid within the tumour microenvironment; our earlier work showed that morphine decreased matrix metalloprotease (MMP)-9 production by breast cancer cells co-cultured with either macrophages or endothelial cells^24^. MMP-9 plays a key role in tumour pathogenesis and progression and triggers the angiogenic switch during carcinogenesis^25^. One of the mechanisms of action for MMP-9 is via extra cellular matrix degradation and release of bioactive VEGF-A^25^,^26^. Our present results show that morphine prevents VEGF mRNA synthesis, rendering unlikely (but not ruling out) that decreased MMP-9-mediated release of VEGF from the extracellular matrix contributes to the decreased VEGF concentration in the conditioned media of morphine-treated, co-cultured cells. Furthermore, the effect of morphine on co-culture increase in MMP-9 was reversed by naloxone^17^, while our current results show that the effect of morphine on co-culture increase in VEGF mRNA is not, suggesting that different mechanisms are at play. Another mode of action for morphine pertinent to the tumour microenvironment is the modulation of the tumour associated macrophage (TAM)-like activation of macrophages. Our previous results show that morphine decreases the alternate activation/M2 polarization of cultured macrophage cells exposed to either IL-4, the prototypical alternate activation-inducing cytokine, or to paracrine stimulation by breast cancer cells^17^. TAMs are known to promote angiogenesis and lymphangiogenesis^26^,^27^, suggesting yet another mechanism by which morphine may prevent these processes.

The effect of morphine on the expression of VEGF in the co-culture of breast cancer cells with macrophages could be a consequence of TNF-α activation. TNF-α has been reported to increase VEGF production in retinal pigment epithelial cells^28^,^29^ and in macrophages^21^. Our experiments show only partial inhibition of VEGF induction by TNF-α neutralizing antibody, with no statistical significance, indicating TNF-α is not the sole/major regulator of VEGF production in co-cultures of breast cancer cells with macrophages. IL-6 has also been shown to induce VEGF expression in cancer cell lines^20^,^30^,^31^. The effect of IL-6 was unaffected by the removal of the hypoxia responsive element from the VEGF promoter^20^. This is in agreement with our results, indicating that the increase in VEGF elicited by macrophage – cancer cell paracrine interaction is secondary to increased IL-6 production in the absence of hypoxia.

Relying on *in vitro* cell-cell communication models to unveil how epithelial and stromal crosstalk promotes cancer (and how morphine prevents this) is both a strength and a limitation of our study. Cell lines grown in monoculture underlie the vast majority of studies on signalling pathways and therapeutic responses relevant to cancer research. In comparison, co-culture systems are advantageous in gene expression studies, and they better model the behaviour of tumour cells in relation to their microenvironment^32^. Co-cultures further allow incremental increases in system complexity so that interactions between specific cell types can be dissected out, and superior control of the experimental parameters compared with an *in vivo* interaction. However, such *in vitro* studies need integration with *in vivo* preclinical models and human tissue studies to translate into valuable understanding of the tumour microenvironment with therapeutic potential^32^.

Overall, our experiments reveal reciprocate proangiogenic interaction between macrophages and breast cancer cells, where both cell types increase, in a non-opioid receptor mediated fashion, their VEGF production, which in turns promotes capillary like tube formation. This is toned down by morphine, as is the basal production of TNF-α and IL-6. Our results further indicate that the change in VEGF induction may be secondary to reduced IL-6 production in the presence of morphine. Together, these results point to a new mechanism by which morphine may affect the tumour micro-environment and regulate tumour growth and metastasis.

## Materials and Methods

### Materials

Cell culture medium, serum and supplements were from Life Technologies (Mulgrave, VIC, Australia). Morphine and naloxone were from Hospira (Mulgrave, VIC, Australia). Matrigel (Basement membrane matrix) was from *In Vitro* Technologies (Noble Park, VIC, Australia). The mouse angiogenesis antibody array was purchased from Abcam (Melbourne, VIC, Australia). The ELISA kits were purchased from Elisa.com (Scoresby, VIC, Australia). Anti-murine IL-6 and anti-murine TNF-α antibodies were from Lonza (Mount Waverley, VIC, Australia) and antimurine VEGF antibody was from R&D Systems (Noble Park, VIC, Australia). AlamarBlue was from Thermo Fisher Scientific (Scoresby, VIC, Australia). Other reagents were purchased from Sigma-Aldrich (Castle Hill, NSW, Australia).

### Cell culture

Mouse RAW264.7 macrophages were grown in Dulbecco’s modified Eagle’s medium (DMEM) supplemented with 10% (v/v) foetal bovine serum (FBS), penicillin (100 units/ml) and streptomycin (100 μg/ml). Murine mammary breast carcinoma cells (4T1) were grown in Roswell Park Memorial Institute medium (RPMI-1640) with 5% FBS (v/v), 1% sodium pyruvate (v/v), penicillin (100 units/ml) and streptomycin (100 μg/ml). Bovine aortic endothelial cells (BAEC) were maintained in Dulbecco’s Modified Eagle Medium: Nutrient Mixture F-12 (DMEM/F12) containing 5% FBS, penicillin (100 units/ml) and streptomycin (100 μg/ml). All cells were incubated in a humidified atmosphere at 37 °C with 5% CO_2_.

### Co-cultures

4T1 and RAW264.7 cells were seeded in equal numbers (1 × 10^5^ cells) in a 12-well plate transwell system, separated by a porous polycarbonate membrane filter (0.4 μM pore size, Corning, NSW, Australia) in a 1:1 mix of complete medium of each cell line and incubated overnight. The medium was replaced with a 1:1 mix of serum-free DMEM and RPMI media, and cells treated with various concentrations of morphine sulfate for 48 h. The cell lysates of RAW264.7 and 4T1 cells and conditioned media were collected.

### AlamarBlue cell proliferation assay

BAEC (5,000 cells/well) were seeded in a 96-well plate in 100 μl serum-containing DMEM/F12 and incubated for 24 h at 37 °C. The cells were washed with PBS twice and placed in 100 μl of either unconditioned medium (DMEM/F12) or conditioned media from 4T1 and RAW264.7 cultured individually or together in the presence or absence of 10 μM or 20 μM morphine. Negative control wells with media but no cells were included. After 48 h, a volume of 10 μl AlamarBlue reagent was added and cells incubated for another 4 h. The absorption at 570 nm and 600 nm was then measured using a SPECTROstar Nano absorbance microplate reader. The calculation of the % of AlamarBlue reduction was performed according to the manufacturer’s protocol:


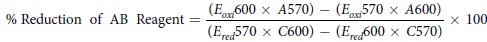


where E_oxi_ and E_red_ are molar extinction coefficient (E) of oxidized AB reagent at 570 and 600 nm, respectively. A570 and A600 represent absorbance of test wells at 570 and 600 nm, respectively. C570 and C600 represent absorbance of negative control at 570 and 600 nm, respectively. Replicate determinations were performed on samples from three independent experiments (N = 3).

### MTT cell proliferation assay

Cell proliferation was evaluated using the 3-(4,5-Dimethylthiazol-2-yl)−2,5-diphenyltetrazolium bromide (MTT) assay. BAEC (5,000 cells/well) were seeded for 24 h and treated with unconditioned or conditioned media for 48 h as above. Cells were then incubated in 100 μl of MTT-containing medium (0.5 mg/mL MTT in serum-free medium) at 37 °C for an additional five hours. The medium was removed and the formazan crystals in cells were dissolved in 100 μl of DMSO. Absorbance was measured at 595 nm with an Imark plate reader (BioRad, USA). The results are expressed as percent of the viability of control cells from replicate determinations from three independent experiments (N = 3).

### Tube formation

Matrigel matrix (60 μl) was loaded into each well of a 96 well plate and incubated to polymerize at 37 °C for 45 min. BAECs (5 × 10^4^ cells) were added on matrigel pre-coated wells in 100 μl of the conditioned medium from either RAW264.7 cells grown individually, 4T1 cells grown individually, or 4T1 and RAW264.7 co-cultured in the presence or absence of morphine. BAECs were incubated for 4–6 h at 37 °C. The formation of capillary-like tubules was documented using a phase-contrast microscope and the number of branching points was counted^33^. Results are presented as the number of branching points forming in conditioned media (mean ± S.D.).

### Mouse angiogenesis antibody array

Membranes were placed in a 2-well plate in blocking buffer for 30 min at room temperature according to the manufacturer’s protocol. The membranes were incubated overnight at 4 °C with equal protein amount of the conditioned medium of 4T1 cells and RAW264.7 cells co-cultured in the presence or in the absence of morphine. Co-culture conditioned medium used in each experiment was a mix of three separate experiments, and the array assay was performed three times (so that a total of 9 separate co-culture conditioned media were employed). The membranes were rinsed thrice with washing buffer I and twice with washing buffer II before incubation with Biotin-Conjugated Anti-Cytokine antibodies (1 ml/well) at 4 °C overnight. After washing the membranes, antibodies were detected using HPR-Conjugated Streptavidin by chemiluminescence. The images were captured using a ChemiDoc Touch Imaging System (Bio-Rad Laboratories Inc.). Densitometric analysis was performed using Image J software.

### Enzyme-linked immunosorbent assay (ELISA)

The concentrations of VEGF-A, TNF-α and IL-6 in conditioned media were determined using mouse ELISA kits (Elisakit, Melbourne, Australia) according to the manufacturer’s instructions. For quantification, a calibration curve of TNF-α, IL-6 or VEGF-A standards was generated. The concentration of the proteins of interest in the samples was calculated using the log–log regression equation of the best fit curve in the graph. Results are presented as mean concentration of the protein ± S.D.

### Quantitative RT-PCR

Total RNA was isolated from the cells and purified using the PureLink RNA Mini Kit (Ambion; Life Technologies, VIC, Australia). RNA (1000–2000 ng) was reverse transcribed using the High-Capacity cDNA Reverse Transcription Kit (Life Technologies, VIC, Australia) and amplified using TaqMan Fast Universal PCR Master Mix (Life Technologies, VIC, Australia) with AmpliTaq Gold DNA Polymerase, and TaqMan Gene Expression Assay VEGF-A (Mm00437306_m1) in a StepOnePlus 7500 real time PCR system (Applied Biosystems, Carlsbad, CA, USA). Quantification was performed relative to 18S ribosomal RNA using the comparative critical threshold (Ct) method^34^ and relative expression of the target gene measured in at least three separate experiments was compared to that of the appropriate control.

### Statistical analysis

Results are shown as mean ± S.D. of at least three separate experiments and were analysed using Graphpad Prism software (version 6). Differences among groups were analysed using one tailed non parametric Mann-Whitney test and statistical significance was considered at P = 0.05.

## Additional Information

**How to cite this article**: Khabbazi, S. *et al*. Morphine decreases the pro-angiogenic interaction between breast cancer cells and macrophages *in vitro.* *Sci. Rep.* **6**, 31572; doi: 10.1038/srep31572 (2016).

## References

[b1] Liang W. & Ferrara N. The complex role of neutrophils in tumor angiogenesis and metastasis. Cancer Immunology Research 4, 83–91 (2016). 26839309 10.1158/2326-6066.CIR-15-0313

[b2] Ferrara N. & Adamis A. P. Ten years of anti-vascular endothelial growth factor therapy. Nat Rev Drug Discov advance online publication (2016). 10.1038/nrd.2015.17 26775688

[b3] Forsythe J. A. . Activation of vascular endothelial growth factor gene transcription by hypoxia-inducible factor 1. Molecular and Cellular Biology 16, 4604–4613 (1996). 8756616 10.1128/mcb.16.9.4604 PMC231459

[b4] Hlatky L., Tsionou C., Hahnfeldt P. & Coleman C. N. Mammary fibroblasts may influence breast tumor angiogenesis via hypoxia-induced vascular endothelial growth factor up-regulation and protein expression. Cancer Res 54, 6083–6086 (1994). 7525053

[b5] Verdelli C. . Tumour-associated fibroblasts contribute to neoangiogenesis in human parathyroid neoplasia. Endocrine-Related Cancer 22, 87–98 (2015). 25515730 10.1530/ERC-14-0161

[b6] Jehs T., Faber C., Juel H. B. & Nissen M. H. Astrocytoma cells upregulate expression of pro-inflammatory cytokines after co-culture with activated peripheral blood mononuclear cells. APMIS 119, 551–561, 10.1111/j.1600-0463.2011.02773.x [doi] (2011). 21749456

[b7] Afsharimani B., Cabot P. & Parat M. O. Morphine and tumor growth and metastasis. Cancer and metastasis reviews 30, 225–238 (2011). 21267766 10.1007/s10555-011-9285-0

[b8] Afsharimani B., Doornebal C. W., Cabot P. J., Hollmann M. W. & Parat M. O. Comparison and analysis of the animal models used to study the effect of morphine on tumour growth and metastasis. British Journal of Pharmacology 172, 251–259 (2015). 24467261 10.1111/bph.12589 PMC4292942

[b9] Gupta K. . Morphine stimulates angiogenesis by activating proangiogenic and survival-promoting signaling and promotes breast tumor growth. Cancer Res 62, 4491–4498 (2002). 12154060

[b10] Farooqui M. . COX-2 inhibitor celecoxib prevents chronic morphine-induced promotion of angiogenesis, tumour growth, metastasis and mortality, without compromising analgesia. Br. J Cancer 97, 1523–1531 (2007). 17971769 10.1038/sj.bjc.6604057 PMC2360252

[b11] Nguyen J. . Morphine stimulates cancer progression and mast cell activation and impairs survival in transgenic mice with breast cancer. Br J Anaesth 113 Suppl 1, i4–13, aeu090 [pii];10.1093/bja/aeu090 [doi] (2014). 24861561 10.1093/bja/aeu090 PMC4111281

[b12] Koodie L., Ramakrishnan S. & Roy S. Morphine suppresses tumor angiogenesis through a HIF-1alpha/p38MAPK pathway. Am J Pathol 177, 984–997 (2010). 20616349 10.2353/ajpath.2010.090621 PMC2913371

[b13] Koodie L. . Morphine inhibits migration of tumor-infiltrating leukocytes and suppresses angiogenesis associated with tumor growth in mice. Am J Pathol 184, 1073–1084, S0002-9440(14)00024-8 [pii];10.1016/j.ajpath.2013.12.019 [doi] (2014). 24495739 10.1016/j.ajpath.2013.12.019 PMC3969995

[b14] Doornebal C. W. . Morphine does not facilitate breast cancer progression in two preclinical mouse models for invasive lobular and *HER2*^+^ breast cancer. Pain 156, 1424–1432 (2015). 25734987 10.1097/j.pain.0000000000000136

[b15] Balasubramanian S. . Morphine sulfate inhibits hypoxia-induced vascular endothelial growth factor expression in endothelial cells and cardiac myocytes. J. Mol. Cell Cardiol 33, 2179–2187 (2001). 11735263 10.1006/jmcc.2001.1480

[b16] Roy S. . Morphine inhibits VEGF expression in myocardial ischemia. Surgery 134, 336–344 (2003). 12947338 10.1067/msy.2003.247

[b17] Khabbazi S., Goumon Y. & Parat M. O. Morphine modulates interleukin-4- or breast cancer cell-induced pro-metastatic activation of macrophages. Scientific Reports 5, 11389 (2015). 26078009 10.1038/srep11389 PMC4468425

[b18] Lee Y. J. . Serum and urine concentrations of morphine and morphine metabolites in patients with advanced cancer receiving continuous intravenous morphine: an observational study. BMC Palliative Care 14, 1–6 (2015). 26507979 10.1186/s12904-015-0052-9 PMC4624671

[b19] Netriova J. . HPLC determination of morphine, morphine-3-glucuronide and morphine-6-glucuronide in human serum of oncological patients after administration of morphine drugs. Pharmazie 61, 528–534 (2006). 16830402

[b20] Wei L. H. . Interleukin-6 promotes cervical tumor growth by VEGF-dependent angiogenesis via a STAT3 pathway. Oncogene 22, 1517–1527 (2003). 12629515 10.1038/sj.onc.1206226

[b21] Lu P. . Critical role of TNF-α-induced macrophage VEGF and iNOS production in the experimental corneal neovascularization. Investigative Ophthalmology & Visual Science 53, 3516–3526 (2012). 22570350 10.1167/iovs.10-5548

[b22] Folkman J., Hahnfeldt P. & Hlatky L. Cancer: looking outside the genome. Nat Rev Mol Cell Biol 1, 76–79 (2000). 11413493 10.1038/35036100

[b23] Martin J. L., Charboneau R., Barke R. A. & Roy S. Chronic morphine treatment inhibits LPS-induced angiogenesis: implications in wound healing. Cell Immunol 265, 139–145 (2010). 20843508 10.1016/j.cellimm.2010.08.002 PMC2950783

[b24] Afsharimani B. . Morphine and breast tumor metastasis: the role of matrix-degrading enzymes. Clin Exp Metastasis 31, 149–158 (2014). 24072419 10.1007/s10585-013-9616-3

[b25] Bergers G. . Matrix metalloproteinase-9 triggers the angiogenic switch during carcinogenesis. Nat. Cell Biol 2, 737–744 (2000). 11025665 10.1038/35036374 PMC2852586

[b26] Kzhyshkowska J. . Role of tumour associated macrophages in tumour angiogenesis and lymphangiogenesis. Frontiers in Physiology 5 (2014). 10.3389/fphys.2014.00075 PMC3942647 24634660

[b27] Coffelt S. B., Hughes R. & Lewis C. E. Tumor-associated macrophages: Effectors of angiogenesis and tumor progression. Biochimica et Biophysica Acta (BBA) - Reviews on Cancer 1796, 11–18 (2009). 19269310 10.1016/j.bbcan.2009.02.004

[b28] Terasaki H. . TNF-α decreases VEGF secretion in highly polarized RPE cells but increases it in non-polarized rpe cells related to crosstalk between JNK and NF-κB pathways. PLos One 8, e69994 (2013). 23922887 10.1371/journal.pone.0069994 PMC3726732

[b29] Wang H., Han X., Wittchen E. S. & Hartnett M. E. TNF-α mediates choroidal neovascularization by upregulating VEGF expression in RPE through ROS-dependent β-catenin activation. Mol Vis 22, 116–128 (2016). 26900328 PMC4736754

[b30] Tzeng H. E. . Interleukin-6 induces vascular endothelial growth factor expression and promotes angiogenesis through apoptosis signal-regulating kinase 1 in human osteosarcoma. Biochemical Pharmacology 85, 531–540 (2013). 23219526 10.1016/j.bcp.2012.11.021

[b31] Huang S. P. . Interleukin-6 increases vascular endothelial growth factor and angiogenesis in gastric carcinoma. J Biomed. Sci 11, 517–527, 10.1159/000077902[doi];77902 [pii] (2004). 15153787

[b32] Casbas-Hernandez P., Fleming J. M. & Troester M. A. Gene expression analysis of *in vitro* cocultures to study interactions between breast epithelium and stroma. Journal of Biomedicine and Biotechnology 2011, 520987, 10.1155/2011/520987 (2011). 22203785 PMC3238808

[b33] Staton C. A., Reed M. W. & Brown N. J. A critical analysis of current *in vitro* and *in vivo* angiogenesis assays. Int. J Exp. Pathol 90, 195–221 (2009). 19563606 10.1111/j.1365-2613.2008.00633.x PMC2697546

[b34] Schmittgen T. D. & Livak K. J. Analyzing real-time PCR data by the comparative CT method. Nat. Protocols 3, 1101–1108 (2008). 18546601 10.1038/nprot.2008.73

